# Supplementary material for: Size-Based Effects of Anthropogenic Ultrafine Particles on Lysosomal TRPML1 Channel and Autophagy in Motoneuron-like Cells
Source: Int J Mol Sci. 2022 Oct 27;23(21):13041. doi: 10.3390/ijms232113041 (PMC9656695; doi:10.3390/ijms232113041)
Supplement: Supplementary file 1 [file ijms-23-13041-s001.zip › ijms-1979110-supplementary.pdf]

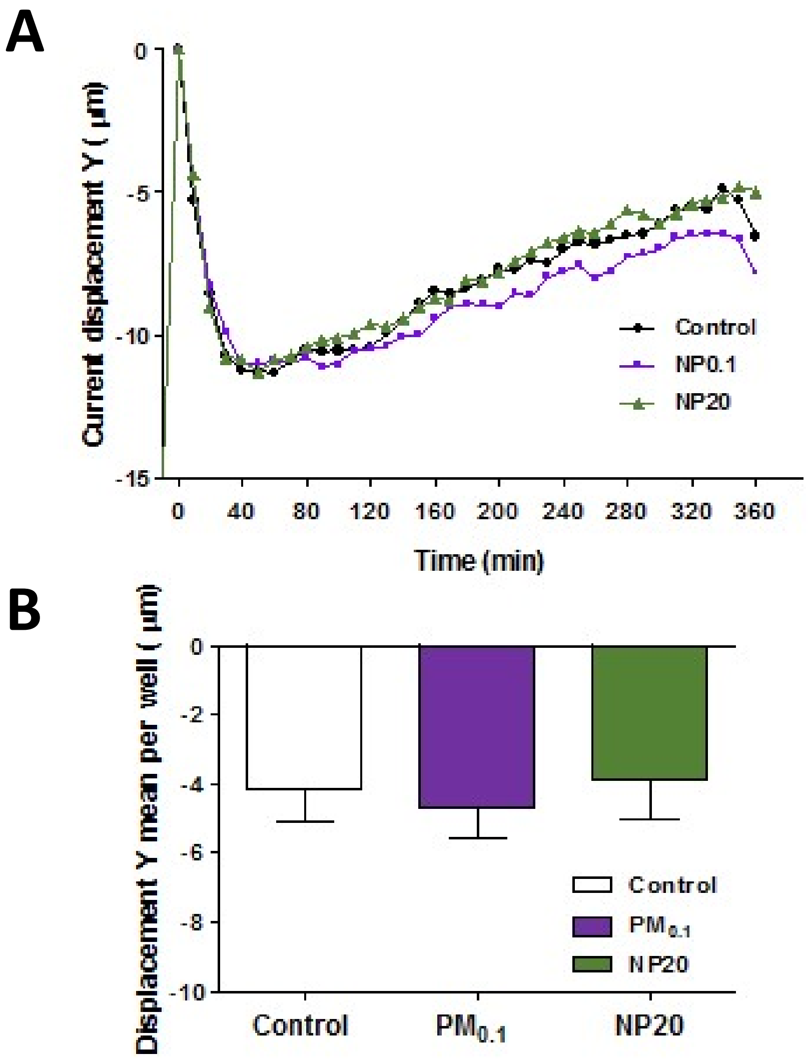
A
0
-5
-10
-15
Current displacement Y (µm)
0
40
80
120
160
200
240
280
320
360
Time (min)
Control
NP0.1
NP20
B
Displacement Y mean per well (µm)
0
-2
-4
-6
-8
-10
Control
PM0.1
NP20
Control
$PM_{0.1}$
NP20

Figure S1

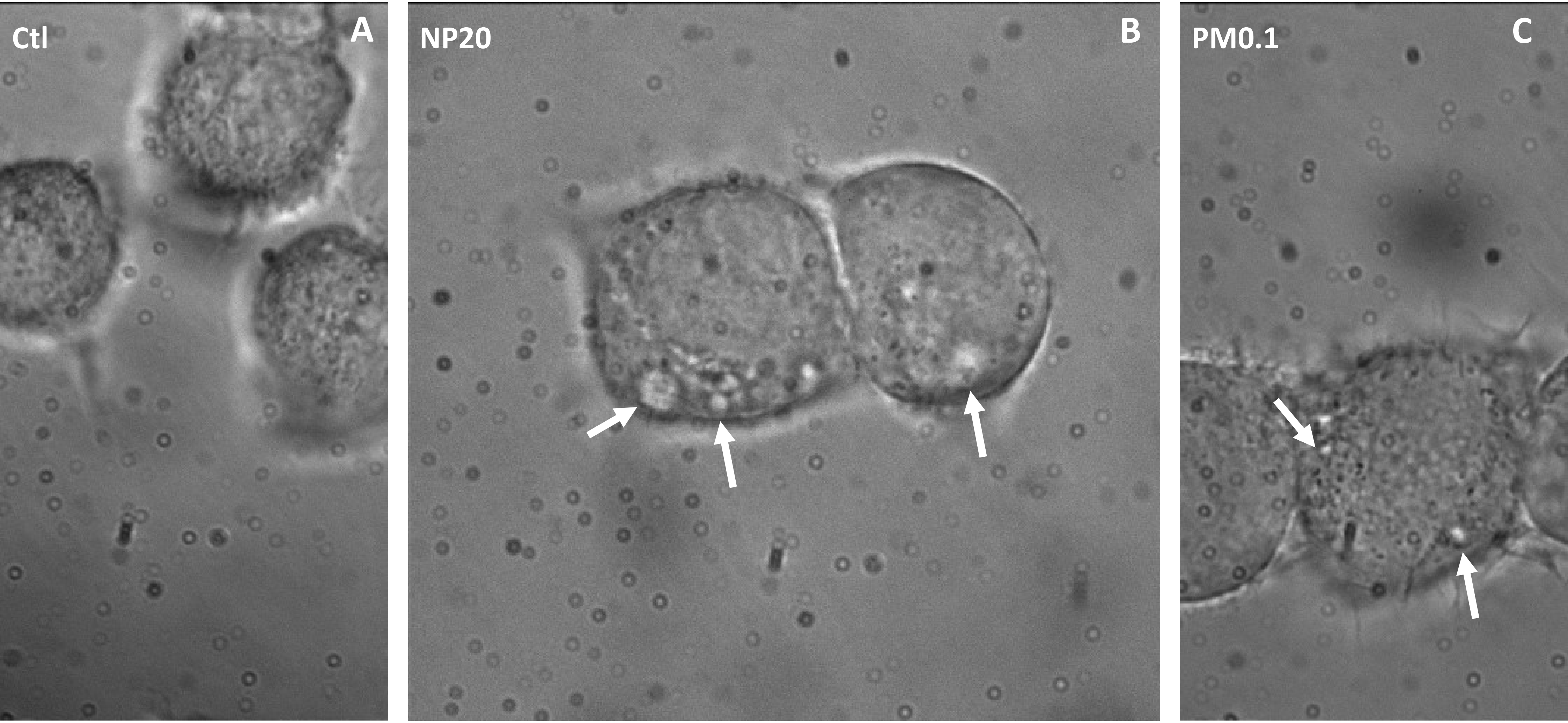

Figure S2

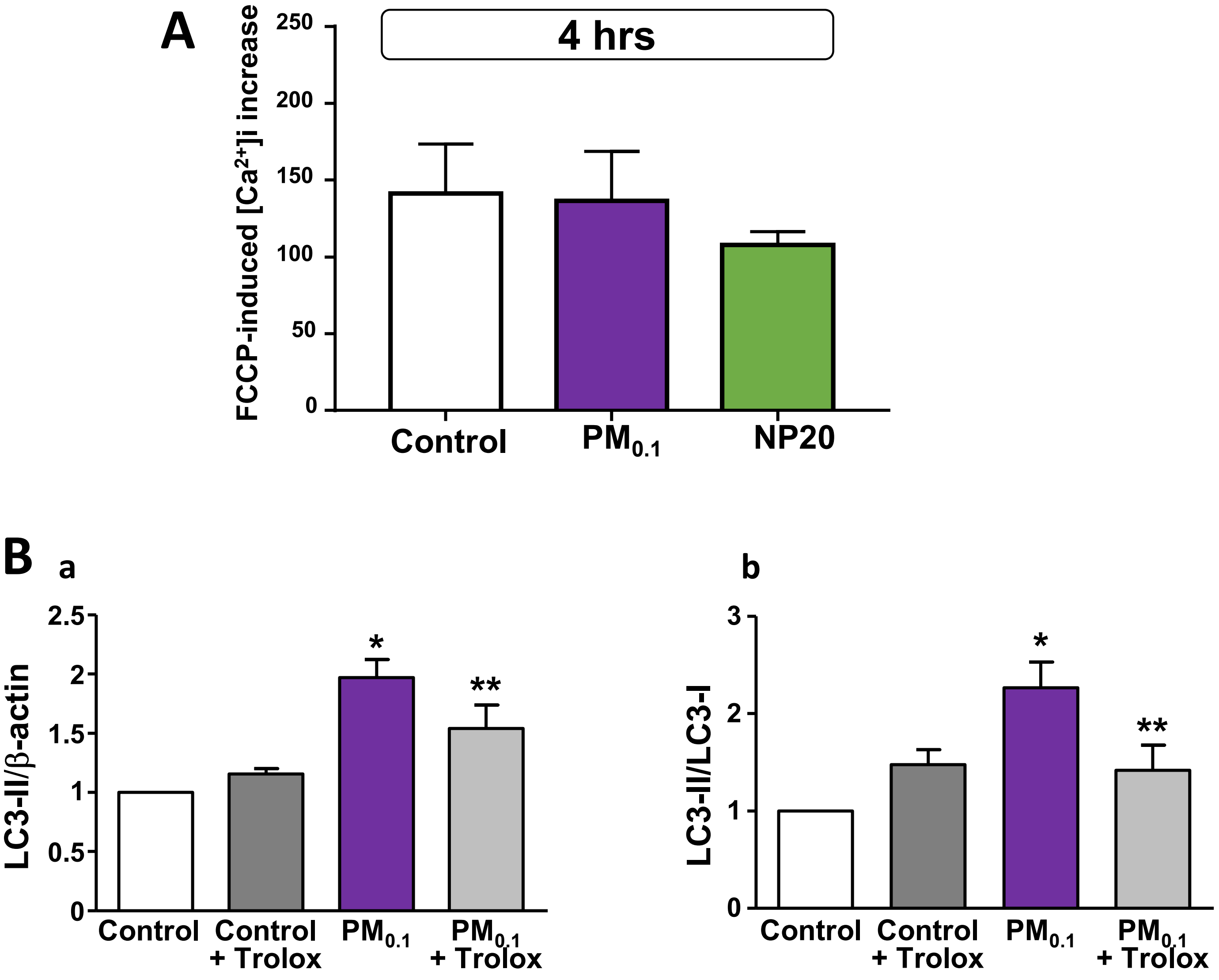
A
4 hrs
FCCP-induced [Ca2+]i increase
250
200
150
100
50
0
Control
PM0.1
NP20
B
a
LC3-II/β-actin
2.5
2
1.5
1
0.5
0
*
**
Control
Control + Trolox
PM0.1
PM0.1 + Trolox
b
LC3-II/LC3-I
3
2
1
0
*
**
Control
Control + Trolox
PM0.1
PM0.1 + Trolox

Figure S3

Figure S1 Effects of PM0.1 and NP20 on the current displacement Y. **A-B** Tracking characteristics (current displacement Y in A, and displacement Y mean per well in B. NSC-34 (150 x 10^3 cells/well) were stimulated (6 hours, 37° C) with DMEM alone (Control), PM0.1 (2,86 ppm) and NP20 (0,71 ppm). The incubation time was carried out in time-lapse and high-content microscopy Operetta High-Content Imaging System (PerkinElmer) per well.

Figure S2 Endocytosis in NSC-34 motor neurons exposed to PM0.1 and NP20. **A-C** Representative images in brightfield of NSC-34 (150 x 10^3 cells/well) exposed for 3 hours to PM0.1 (2,86 ppm) and NP20 (0,71 ppm).

Figure S3 Effect of PM0.1 and NP20 on mitochondrial dysfunction and LC3 in NSC-34 motor neurons. A Bar graph representing the quantification of the effect of acute exposure to FCCP on [Ca2+]i after treatment with PM0.1 and NP20 for 4 hours. For each group at least n=15 cells were detected. **B** Representative quantification of LC3-I/LC3-II expression in NSC-34 motor neurons exposed to PM0.1 (48 hrs) in the presence of trolox. Each bar represents the mean ± S.E. of data obtained from three different sessions. *$p < 0.01$ vs control, **$p<0.01$ vs PM0.1 alone.
